# Lack of Temporal Impairment in Patients With Mild Cognitive Impairment

**DOI:** 10.3389/fnint.2019.00042

**Published:** 2019-09-12

**Authors:** Giovanna Mioni, Lucia Meligrana, Francesco Perini, Michela Marcon, Franca Stablum

**Affiliations:** ^1^Dipartimento di Psicologia Generale, Università di Padova, Padua, Italy; ^2^U.O. Neurologia e Geriatria Ospedale San Bortolo, Vicenza, Italy

**Keywords:** mild cognitive impairment, time processing, time bisection, emotion, temporal dysfunction

## Abstract

In the present study, we investigate possible temporal impairment in patients with mild cognitive impairment (MCI) and the amount of temporal distortions caused by the presentation of emotional facial expressions (anger, shame, and neutral) in MCI patients and controls. Twelve older adults with MCI and 14 healthy older adults were enrolled in the present study. All participants underwent a complete neuropsychological evaluation. We used three timing tasks to tap temporal abilities, namely time bisection (standard intervals lasting 400 and 1600 ms), finger-tapping (free and 1 s), and simple reaction-time tasks. The stimuli used in the time bisection task were facial emotional stimuli expressing anger or shame to investigate a possible contribution of emotional information as previously observed in healthy adults. MCI patients showed temporal abilities comparable to controls. We observed an effect of facial emotional stimuli on time perception when data were analyzed in terms of proportion of long responses, and this result was mainly driven by the temporal overestimation when a facial expression of anger was presented in controls. Results seem to suggest that the severity of the cognitive dysfunction accounts more for subjective temporal impairment than a compromised internal clock.

## Introduction

Many daily life activities rely on accurate temporal estimation, and indeed, time processing represents a fundamental cognitive function. According to internal clock models of time processing (Gibbon et al., 1984), the raw temporal material comes from the number of pulses emitted by a pacemaker (internal clock) and stored in an accumulator during the event to be timed; duration judgments are made by comparing the number of pulses counted for the observed interval with a value previously stored in memory. Finally, a decision process compares the current duration values with those in working and reference memory to decide on the adequate temporal response. An extension of this model comes from the attentional gate model (Zakay and Block, 1996), developed to explain the influence of a person’s attentional resource allocation on temporal judgments. An attentional gate is positioned before the accumulator and regulates the flow of pulses from the pacemaker; when attentional resources are allocated to timing, the gate is opened wider, allowing for more pulses entering the accumulator, resulting in more accurate temporal judgments (Zakay and Block, 1996).

Errors in temporal processing may depend on different factors and occur at each stage of the internal clock model. Variations in the rate of pulses’ emission by the pacemaker are often reported to be an important cause of temporal errors. These variations have several causes, like changes in body temperature (Aschoff, 1998; Mioni et al., 2016a), experiencing emotions (Gil and Droit-Volet, 2011), and using pharmacological substances (Rammsayer, 2008; Coull et al., 2011). As mentioned, according to the attentional gate model (Zakay and Block, 1996), the gate is the part of the model that is directly associated with the mechanisms of attention. When the gate is closed, the pulses that are emitted by the pacemaker are accumulated in the counter. Indeed, it is the amount of attention paid to time that determines the accumulation of pulses in the counter. The demonstration of the role of attention in temporal processing is often based on the dual-task paradigm, in which attention has to be divided between temporal and non-temporal tasks. Results showed that when more attention is dedicated to time, more pulses are accumulated in the counter, and fewer temporal errors are produced (Block et al., 2010). When participants are asked to estimate time while performing other cognitive tasks, the accuracy of time estimation is reduced because time estimation shares attentional resources with the non-temporal tasks and the amount of the shared resources depends on the nature of the second task (Brown, 1997).

Different brain areas have been identified to play a critical role in temporal processing (Rubia and Smith, 2004; Merchant et al., 2013; Muller and Nobre, 2014; Paton and Buonomano, 2018). In particular, the integrity of the right dorsolateral prefrontal cortex and right inferior parietal lobe has been shown to be necessary for processing temporal intervals of several seconds (Lewis and Miall, 2003; Mioni et al., 2014). The importance of the cerebellum in timing processes is also well established. Patients with cerebellar lesions showed poor performance on both motor tapping and time estimation tasks, in the range of both hundreds of milliseconds and a few seconds (Rubia and Smith, 2004; Gooch et al., 2010). The role of the basal ganglia in time estimation and motor timing functions is confirmed by studies with Parkinson’s disease (PD) patients showing deficits in time perception that can be improved with dopaminergic treatments (Meck et al., 2008; Jones and Jahanshahi, 2014).

However, it should be noted that most of the brain areas and networks involved in temporal processing are also critically involved in other cognitive functions, such as attention, working memory, or motor control (Matell et al., 2005; Üstün et al., 2017).

It is, therefore, important to ascertain whether the impairment in temporal processing often displayed by patients with cognitive dysfunctions truly reflects a deficit in one of the stages involved in temporal processing (i.e., compromised internal clock) or whether it is just a consequence of a general impairment in the cognitive functions required to process time (i.e., attention or working memory).

As an example, consider the patients with traumatic brain injury (TBI) who exhibit sensorimotor, behavioral, and cognitive sequelae, which may include deficits of attention, speed of processing, memory, planning and problem solving, and lack of self-awareness (Lezak, 2004). Mioni et al. (2014) reviewed studies conducted to investigate temporal abilities in TBI and reported temporal misperception and higher variability for TBI patients than for controls. But, temporal dysfunctions in TBI patients were mainly related to deficits in cognitive functions involved in temporal processing (i.e., working memory, attention, and executive functions) rather than to an impairment in time perception caused by a compromised internal clock. In fact, temporal dysfunctions were observed when the temporal intervals exceeded the working memory span or when the tasks employed required high cognitive functions to be performed. Moreover, higher temporal variability observed in TBI patients is also a sign of impaired frontally mediated cognitive functions that affect temporal representation (Mioni et al., 2014).

The number of patients with cognitive complaints has been rising as a consequence of the increasing aging of the population. Clinicians have especially focused on patients diagnosed with mild cognitive impairment (MCI) because they carry a high risk of developing dementia in the ensuing few years. Previous studies showed compromised time processing in patients with Alzheimer’s disease (El Haj and Kapogiannis, 2016) and the behavioral variant of frontotemporal dementia (Wiener and Coslett, 2008), but very few studies have been conducted to test temporal abilities in MCI patients. Rueda and Schmitter-Edgecombe (2009) first examined temporal abilities in MCI patients using a verbal estimation task with filled short (10 and 25 s) and long (45 and 60 s) intervals. Results showed comparable performance between MCI patients and age-matched controls at both short and long intervals. More recently, Coelho et al. (2016) replicated these findings in verbal estimation and time production tasks (7, 32, and 58 s). The authors then concluded that an abnormal internal clock was not the basis for these alterations in the perception of the subjective passage of time, since the perception of the interval lengths was not different in MCI patients compared to controls. Summing up these results, it seems that there are no temporal dysfunctions in MCI patients. However, a firm conclusion should be driven by more than two studies, which calls for more investigation in this field.

Interestingly, Mioni et al. (2016b) first tested PD patients with or without MCI to investigate the role of cognitive impairment in temporal dysfunction in PD patients. Results showed that PD patients with MCI were more likely to underestimate long and overestimate short temporal intervals than PD patients without MCI and control participants (migration effect; Malapani et al., 1998). The authors concluded that temporal impairment observed in PD patients with MCI was mainly caused by a memory dysfunction rather than variation at the internal clock level (Mioni et al., 2016b). Indeed, according to Malapani et al. (1998), changes in clock speed would not cause any bias in temporal accuracy under conditions in which participants were trained and tested in the same neurological state (“on” or “off” medication). Conversely, memory dysfunction for a previous learned interval (during the training phase, when two temporal intervals are learned) would affect the estimation of the later learned interval, leading to a migration of the two target durations toward each other.

Here we decided to move a step forward in this investigation by testing a group of patients with only MCI symptoms. Specifically, we tested MCI patients during a time bisection task to investigate temporal impairment in these patients. If the results observed by Mioni et al. (2016b) with PD with MCI were mainly driven by their cognitive impairment, we should observe a leftward shift of the psychometric function (indicating temporal overestimation) and higher temporal variability in MCI patients compared to controls; however, this is not what was observed in previous studies (Rueda and Schmitter-Edgecombe, 2009; Coelho et al., 2016). Two finger-tapping tasks (spontaneous rate and 1 s rate) were included considering that spontaneous-tempo measure has been related to the pace of the internal clock (Pouthas and Perbal, 2004; Perbal et al., 2005; Jones and Jahanshahi, 2009). Moreover, a simple reaction-time task was included to control for possible differences in motor performance.

Here, we also investigate the possible contribution of emotional stimuli (implicit elaboration of emotional stimuli) on subjective perceived duration in MCI patients. It is known that the rate of the internal clock, and consequently, the perceived duration, can be influenced by the presentation of emotional stimuli: an increased level of arousal increases the speed of the pacemaker (Droit-Volet and Meck, 2007; Gil and Droit-Volet, 2011). For a given duration, if the pacemaker runs faster, more pulses reach the accumulator, producing temporal overestimation (Droit-Volet et al., 2004; Effron et al., 2006). This has been observed in particular in healthy participants, with emotional pictures generating high arousal, such as facial expressions of anger, fear, happiness, and sadness (Droit-Volet et al., 2004; Droit-Volet and Gil, 2009; Gil and Droit-Volet, 2011; Lee et al., 2011; Mioni et al., 2017).

Taking these together, we investigate if differences in temporal judgments in MCI patients relative to healthy controls are related to the level of cognitive function in MCI patients. It is of great interest to assess cognitive functions as a possible mediator of lower temporal abilities in MCI patients and compare these new data with previous studies testing PD patients with and without MCI to fully understand the cause of temporal impairment in these clinical samples. To characterize the clinical sample and to measure the cognitive level in MCI participants and controls, a neuropsychological evaluation is included. The second aim of the present study is to investigate the effect of emotional facial expression on time perception. We included anger and shame as emotional stimuli, given that they have generated different effects on time perception (Gil and Droit-Volet, 2011). In particular, we predicted an overestimation of temporal intervals when the stimulus marking time represents anger and an underestimation of temporal intervals when the stimulus represents shame. Compared to healthy older adults, MCI patients exhibit worse facial emotion recognition in the detection of negative emotions. These deficits appear to occur in the context of intact facial information processing (McCade et al., 2011). Therefore, here we investigate, for the first time, if the magnitude of temporal distortions caused by the presentation of the facial emotional stimuli would be the same for MCI patients and controls.

## Materials and Methods

### Participants

Twelve MCI patients (6 males) and 14 healthy older adults (7 males) took part in the present study. Demographic and clinical characteristics of MCI patients and controls are reported in Table 1 and the Supplementary Materials. MCI participants were recruited from a major hospital in Vicenza, Italy (San Bortolo Hospital), and selected accordingly to the Alzheimer’s Disease Diagnostic Guidelines of the National Institute on Aging (Albert et al., 2011); specifically, one patient was “single-domain non-amnestic MCI,” three patients “multiple-domain non-amnestic MCI,” four patients “single-domain amnestic MCI,” and four patients “multiple-domain amnestic MCI” (Petersen and Negash, 2008; del Carmen Díaz-Mardomingo et al., 2017). Patients were not hospitalized, but they came to the hospital for screening and controls (see the section “Procedure”). Controls were volunteers from the local community (Vicenza and Padova) and patients’ relatives or friends and were matched to MCI participants on the basis of age and years of education (±2 years with respect to the MCI sample). The Mini-Mental State Examination (MMSE; Folstein et al., 1975) was used to evaluate global cognitive functioning. The total possible score is 30 points; a score of 24 or above is considered within the normal range. The Montreal Cognitive Assessment (MoCA; Santangelo et al., 2015) was used to screen for mild cognitive dysfunction. The total possible score is 30 points; a score of 15.5 or above is considered within the normal range. The Frontal Assessment Battery (FAB; Appollonio et al., 2005) was administrated to evaluate frontal lobe functions; higher scores indicate better performance, and the maximum score is 18.

**TABLE 1:** Mean and standard deviation for descriptive statistics and neuropsychological evaluation for control and MCI groups.

	**Control group, *n* = 14**	**MCI patients, *n* = 12**	
		
**Measure**	***M* (*SD*)**	***M* (*SD*)**	***t***
Age	69.64(6.86)	67.00(5.67)	1.06
**Year of education**	10.21(4.19)	10.17(4.02)	0.03
MMSE	29.21(0.97)	27.75(1.21)	3.41^∗^
MoCA	26.00(1.02)	22.00(2.04)	5.50^∗^
FAB	17.14(0.95)	15.83(1.53)	2.67^∗^
CPM	29.50(3.20)	28.10(3.96)	0.98
**TMT**			
Part A	56.36(19.80)	46.33(12.32)	1.52
Part B	125.86(51.39)	143.08(64.67)	0.76
B−A	69.50(44.70)	96.75(59.19)	1.43
Semantic fluency	41.07(9.60)	36.27(4.63)	1.52
**MCST**			
Category	5.78(0.57)	3.90(1.81)	3.66^∗^
Perseverative errors	1.00(1.71)	2.00(1.94)	1.33
**ROCF**			
Copy	32.71(2.40)	29.50(5.76)	1.91
Delayed recall	18.39(4.65)	19.50(30.48)	0.13
**Word list**			
Immediate recall	38.71(8.67)	33.92(9.18)	1.37
Delayed recall	7.85(2.24)	5.58(3.23)	2.11^∗^
CDT	11.85(2.11)	13.42(1.56)	2.11^∗^

*MMSE, Mini-Mental State Examination; MoCA, Montreal Cognitive Assessment; FAB, Frontal Assessment Battery; CPM, Coloured Progressive Matrices; TMT, Trail Making Test in parts A and B and difference in reaction time between parts B and A (B−A); MCST, Modified Card Sorting Test; ROCF, Rey–Osterrieth complex figure test in immediate recall and delayed recall conditions; word list, word-list recall task in immediate recall and delayed recall conditions; CDT, clock drawing test. ^∗^*p* < 0.05.*

Exclusion criteria for all participants included possible dementia or severe cognitive impairment (as defined by MMSE or MoCA scores below the cutoff), treatment with anticholinergic medications, treatment with certain dopaminergic or benzodiazepine medications known to interfere with cognitive functioning, history of neurosurgery or other neurological conditions, significant history or current psychiatric disorders, and any condition (e.g., depression) that would interfere with testing.

No differences in terms of age (*p* = 0.300) or level of education (*p* = 0.977) were found between groups. MCI patients shower lower scores on the MMSE (*p* = 0.002), MoCA (*p* < 0.001), and FAB (*p* = 0.013) compared to controls (Table 1).

### Procedure

Mild cognitive impairment patients were tested at San Bortolo Hospital, Vicenza, Italy, whereas controls were tested in their own homes in the areas of Vicenza and Padova (Italy). All patients were not hospitalized, and they came to the hospital for screening and controls. During the first visit, patients performed the neuropsychological evaluation; if they met the criteria for a possible evaluation of MCI, they were contacted again by the experimenter and performed the temporal tasks. A maximum of 2 weeks intervened between the first visit (neuropsychological evaluation) and the temporal tasks. Controls were also tested in two experimental sessions.

During the time bisection task, participants were seated at a distance of approximately 60 cm in front of a 15-inch PC monitor screen. E-Prime^®^2.0 was used to program and run the experiment. After the time bisection task, all participants were asked to name the emotional facial expression presented, and all identified the emotions correctly. Afterward, participants performed the finger-tapping tasks (free and 1 s) and the simple time reproduction task.

Informed consent was collected from all participants, and the study was conducted in accordance with the Helsinki Declaration (59th World Medical Association (WMA) General Assembly, Seoul, Republic of Korea, October 2008).

### Materials

#### Neuropsychological Assessment

Neuropsychological tests were included to investigate cognitive abilities in MCI patients and controls.^1^ The neuropsychological evaluation was conducted within 2 weeks before the timing task and included the *Coloured Progressive Matrices* (CPM; Italian normative data in Caffarra et al., 2003) to tap abstract reasoning; the *Trail Making Test* (TMT, parts A and B; Italian normative data in Giovagnoli et al., 1996) for attention and working memory; the *Semantic Fluency* test (Italian normative data in Novelli et al., 1986) and the *Modified Card Sorting Test* (MCST; Italian normative data in Caffarra et al., 2004) for executive functions; the *Rey–Osterrieth Complex Figure Test* (ROCF copy and delayed recall; Italian normative data in Caffarra et al., 2002) and the *word-list recall* task (Italian normative data in Carlesimo et al., 1996) to test memory (immediate and delayed recall); and finally, the *Clock Drawing Test* (CDT; Italian normative data in Caffarra et al., 2011) to test visual–spatial functions.

#### Time Bisection Task

**FIGURE 1:**
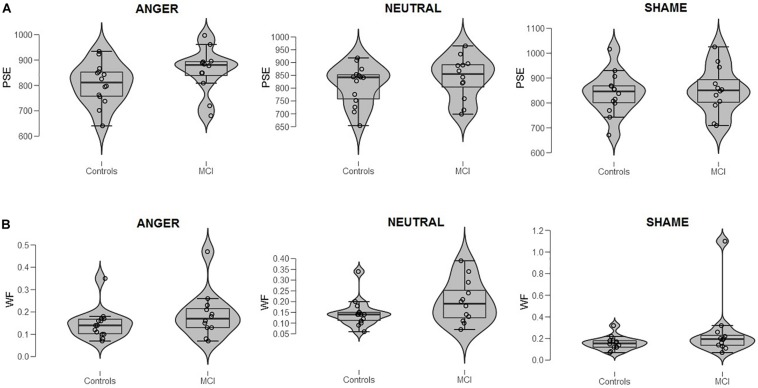
**(A)** Bisection point (BP) and **(B)** Weber ratio (WR) of the time bisection task as a function of *Group* (MCI and control) and *Emotion* (anger, shame, and neutral). Each dot represents a single participant.

The experimental session started with the training phase, in which participants were asked to memorize two standard durations: 400 (short standard) and 1600 ms (long standard), each presented 10 times [see Mioni et al. (2016b) for the same procedure]. During the training phase, the stimulus used for marking time was a gray oval with a size similar to that of the target stimuli. During the testing phase, participants were instructed to judge new temporal intervals and press with their index fingers the keys marked with the label “S” (positioned over the “S” key of a QWERTY keyboard) or “L” (positioned over the “K” key of a QWERTY keyboard) if the new temporal interval was closer to the short standard or to the long standard, respectively. Response keys were counterbalanced between participants. Emotional stimuli were used during the testing phase to mark the temporal intervals and represented male and female faces expressing anger, shame, and neutral emotion. The stimuli were selected from the Montreal Set of Facial Displays of Emotion (Beaupré et al., 2000). Participants were required to perform four blocks with pictures of female facial expressions (Figure 1A) and four blocks with pictures of male facial expressions (Figure 1B). Within each block, 12 pictures of females or males were randomly presented for each of the comparison durations (400, 600, 800, 1000, 1200, 1400, and 1600 ms), for a total of 84 stimuli.

#### Finger-Tapping Task

In the finger-tapping task (Perbal et al., 2005), participants were required to tap as regularly as possible at the pace they preferred (spontaneous tempo) or at a 1 s pace (1 s tempo). Both the beginning and the end of a session were marked by a cross at the center of the computer screen. Participants were instructed to tap when they saw the cross and continue until the cross disappeared. All participants performed the spontaneous finger-tapping task before the 1-s-paced finger-tapping task.

#### Simple Reaction-Time Task

In the simple reaction-time task (Perbal et al., 2005), participants were required to press a designed key as fast as possible in response to a stimulus (gray circle) that appeared in the center of the computer screen at a random inter-stimulus onset interval (IOI) ranging from 2000 to 3000 ms (long IOI condition) and from 1000 to 2000 ms (short IOI condition). All subjects performed the long IOI condition before the short IOI condition, each of which contained 35 trials.

### Statistical Analyses

To explore the effect of emotional facial expression on perceived duration in MCI patients and controls, we analyzed the proportion of “long” responses for each stimulus duration, and these were included in an ANOVA with *Group* (MCI and controls) as a between-subjects factor and *Emotion* (anger, shame, and neutral) and *Temporal interval* (400, 600, 800, 1000, 1200, 1400, and 1600 ms) as within-subjects factors.^2^

We also calculated two indices, one for the perceived duration and one for sensitivity. For each participant, a seven-point psychometric function was traced, plotting the seven comparison intervals on the *x*-axis and the probability of responding long on the *y*-axis. The cumulative normal function was fitted to the resulting curves. More specifically, we used a non-linear least squares analysis, with a Levenberg–Marquardt algorithm. The first index calculated was the bisection point (BP) (i.e., proportion of stimulus durations judged to be long = 0.50); an observed shift of the BP for the different emotional facial expressions presented can be interpreted as an indicator of differences in these conditions, with smaller BP (left shift of the psychometric function) values meaning longer perceived durations (Kopec and Brody, 2010; Mioni et al., 2016b; Penney and Cheng, 2018). The second dependent variable was the Weber ratio (WR), which is based on difference limen (DL) divided by the BP. We used a maximum-likelihood approach for fitting the psychometric function (Oberfeld et al., 2014). Each fit provided an estimate of the mean of the cumulative normal distribution function (representing the BP), and an estimate of its SD, representing the spread of the psychometric function. We defined the DL as half the difference between the 75% and the 25% point on the psychometric function, which for a cumulative normal psychometric function is given by DL = 0.67449 ^∗^ *SD*. This is a measure of temporal sensitivity: smaller values indicate more sensitive and less variable timing. BP and WR data were included in a repeated-measures ANOVA with *Group* (MCI and controls) as a between-subjects factor and *Emotion* (anger, shame, and neutral) as a within-subjects factor.

The significant analyses were followed by *post hoc* analyses with Bonferroni’s correction to reduce the Type I error rate, and the effect size was estimated with the partial eta-squared index ($\eta_{\text{p}}^{2}$).

Separate *t*-tests were conducted on performances of finger-tapping, simple reaction-time, and neuropsychological tests in MCI patients and controls (Table 1).

## Results

When data were analyzed in terms of proportion of long responses, significant main effects for *Emotion* [*F*(2,48) = 6.23, *p* = 0.004, $\eta_{\text{p}}^{2}$ = 0.21] and *Temporal interval* [*F*(6,144) = 634.02, *p* < 0.001, $\eta_{\text{p}}^{2}$ = 0.96] were found, indicating that participants pressed long more times when anger and neutral were presented compared to shame [anger–shame, *t*(31) = 2.50, *p* = 0.018; neutral–shame, *t*(31) = 2.09, *p* = 0.045]; moreover, participants pressed long more times as standard duration increased. No main effect of *Group* was found (*p* = 0.439, $\eta_{\text{p}}^{2}$ = 0.02), nor other significant interactions (all *p*s ≥ 0.056, $\eta_{\text{p}}^{2}$ ≤ 0.11).

**FIGURE 2:**
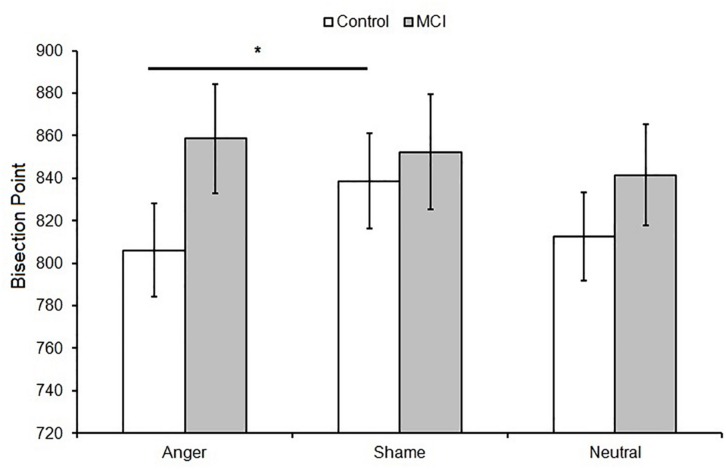
Mean bisection point (BP) as a function of *Group* (MCI and control) and *Emotion* (anger, shame, and neutral). Error bars represent the standard error of the mean. ^∗^ = significant difference *p* < 0.05.

When data were analyzed in terms of BP, the main effect of *Emotion* was significant [*F*(2,48) = 3.40, *p* = 0.041, $\eta_{\text{p}}^{2}$ = 0.12]; moreover, *Group* interacted with *Emotion* [*F*(2,48) = 3.64, *p* = 0.034, $\eta_{\text{p}}^{2}$ = 0.13]. *Post hoc* analyses showed no differences between groups at each level of emotion [anger, *t*(24) = 1.56, *p* = 0.131; shame, *t*(24) = 0.39, *p* = 0.701; and neutral, *t*(24) = 0.915, *p* = 0.369]. A lower BP value was observed when an emotional facial expression of anger was presented compared to sham, indicating temporal overestimation, but this was evident only in the control group (*p* = 0.007, $\eta_{\text{p}}^{2}$ = 0.35) (Figures 1A, 2). No effect of emotions within the MCI group (*p* = 0.243, $\eta_{\text{p}}^{2}$ = 0.12) was found [anger, MCI PSE = 859(89), control PSE = 806(81); shame, MCI PSE = 852(94), control = 839(84); and neutral, MCI PSE = 841(83), control = 812(77)].

When data were analyzed in terms of WR, no main effects of *Group* (*p* = 0.163, $\eta_{\text{p}}^{2}$ = 0.08; MCI = 0.21, control = 0.15), *Emotion* (*p* = 0.164, $\eta_{\text{p}}^{2}$ = 0.07), or interaction were found (*p* = 0.366, $\eta_{\text{p}}^{2}$ = 0.04) (Figure 1B^3^).

No difference between groups was observed for the spontaneous [*t*(24) = 1.50, *p* = 0.146; control = 853(190) ms, MCI = 1148(706) ms] and 1 s [*t*(24) = 1.21, *p* = 0.234; control = 1021(344) ms, MCI = 1278(647) ms] finger-tapping tasks as well as for the simple reaction-time task [*t*(24) = 0.90, *p* = 0.379; control = 331(57) ms, MCI = 354(75) ms].

The MCI group had lower scores on the MCST category (*p* < 0.001), word-list delayed recall (*p* = 0.046), and CDT (*p* = 0.045) but performed like controls on the other neuropsychological tests (all *p* ≥ 0.894) (Table 1).

## Discussion

Temporal processing is one of the fundamental cognitive functions in daily life. Accurate temporal processing requires the proper functioning of the internal clock as well as various cognitive processes, such as attention and memory. Therefore, it has been suggested that patients with reduced attentional and memory abilities would also present temporal dysfunctions (Pouthas and Perbal, 2004; Rubia and Smith, 2004; Allman and Meck, 2011).

Increasing attention has been paid in recent years to monitoring the cognitive spectrum spanning from normal aging to Alzheimer’s disease (Petersen, 2004; Petersen et al., 2009). There is likely a transitional period between normal aging and the diagnosis of clinically probable very early Alzheimer’s disease, and patients in this transitional zone have been described as MCI patients. The most typical MCI patient is one who has memory impairment beyond what is felt to be normal for that age but who is relatively intact in other cognitive domains (Petersen, 2004; Petersen et al., 2009). Despite the role of temporal processing in everyday functioning, few studies have investigated time estimation abilities in persons with MCI. The present study aims to further investigate temporal ability in MCI patients as well as to investigate the possible contribution of emotional stimuli to their temporal judgments.

We did not observe temporal impairment in MCI patients, a result consistent with previous studies (Rueda and Schmitter-Edgecombe, 2009; Coelho et al., 2016), although surprising considering that temporal impairment is often observed in healthy older adults either when they are required to process neutral stimuli (Block and Zakay, 1997; Block et al., 1998; Turgeon et al., 2016; Lamotte and Droit-Volet, 2017) or when temporal intervals are marked by emotional stimuli (Nicol et al., 2013). Indeed, several studies have found age-related differences in time estimation and have related them to either variation at the level of the internal clock or age-related changes in the cognitive processes involved in time estimation. The assumption of the slowing of an internal timing mechanism with aging has been proposed by several authors to account for the slower information processing found in older adults on perceptual and motor tasks (Wearden et al., 1997; Perbal et al., 2002; Pouthas and Perbal, 2004). Furthermore, the slowing of information processing in older adults is often associated with the subjective feeling that time is passing at a faster pace than at younger ages (Fraisse, 1984). However, it is important to consider that variations in temporal processing in healthy older adults are reported when they are compared to younger adults. Here, as well as in the work of Coelho et al. (2016) and Rueda and Schmitter-Edgecombe (2009), MCI patients were compared with matched healthy older adults, and no differences were observed. It is, therefore, possible that older adults, independently of their cognitive abilities, present similar age-related changes at some or all of the different levels of temporal processing (i.e., internal clock). However, it should be noted that MCI patients present reduced cognitive functions compared to healthy older adults. It is possible that our MCI patients were at the first stage of their cognitive decline, and this might explain the lack of temporal differences between MCI patients and controls. Indeed, MCI patients performed like controls in most of the tasks included in the neuropsychological evaluation.

In line with the observation of similar age-related changes at the level of the internal clock in MCI and healthy older adults, we reported comparable performance at both finger-tapping tasks between these two groups. Finger-tapping tasks are often considered good measures of spontaneous tempo and a measure of subjective experience of time (Cellini et al., 2015; Mioni et al., 2016a). Previous studies reported temporal dysfunction in PD patients when tested with finger-tapping tasks (Artieda et al., 1992; Pastor et al., 1992; O’Boyle et al., 1996), claiming an association between performance at a spontaneous tapping task and pace of the internal clock (Rao et al., 1997, 2001). The lack of differences between MCI patients and controls at a finger-tapping task does not exclude a dysfunction at the level of the internal clock but only confirms a similar pattern in both groups.

Mioni et al. (2016b) showed lower temporal performance in PD-MCI patients compared to PD patients without MCI and controls and concluded that temporal impairment was mainly caused by cognitive dysfunctions. Combining our new results with the findings of Mioni et al. (2016b), it seems possible to conclude that more severe cognitive impairment accounts for temporal dysfunction more than variation at the internal clock level. Future studies should test how different gradients of cognitive impairments modulate temporal performance.

Regarding the effect of emotion on time perception, healthy controls showed a longer BP value for anger compared to shame facial emotional expression, a finding that is consistent with previous research indicating temporal overestimation when highly arousing stimuli are presented (Droit-Volet et al., 2004; Effron et al., 2006). No effect of emotional stimuli was observed in MCI patients. McCade et al. (2011), in their 2011 review, reported worse facial emotion recognition in MCI patients compared to healthy older adults. Although no consistent emotion-specific impairment was reported, there was some evidence to suggest that the detection of negative emotions was selectively affected. This may reflect the employment of mainly negative emotions in the studies included in their review. Support for emotional recognition impairment in MCI patients comes from neuroimaging studies demonstrating atrophy in regions implicated in emotion processing, including the amygdala and fusiform gyrus (Whitwell et al., 2007), superior temporal gyrus and insula (Karas et al., 2004), and anterior cingulate (Chételat et al., 2002).

A limitation of the present study comes from the sample size. We acknowledge that the two groups are quite small, but we believe that our study can still provide interesting insights into the understanding of temporal dysfunction in MCI patients as well as on the effect of facial emotional recognition in MCI patients and controls.

To conclude, the present study adds new insight into the study of temporal processing in MCI patients as well as in PD patients. The results showed comparable temporal ability in MCI patients and controls, suggesting a similar pattern of temporal dysfunction in both groups. Considering the present results and those of Mioni et al. (2016b), it seems that the severity of the cognitive dysfunction can account more for subjective temporal impairment than a compromised internal clock; however, this conclusion must be taken with caution, and future studies should include control groups of different ages (i.e., young adults and younger older adults) and with different gradients of cognitive decline. This will help in understanding the progression of cognitive decline and the subsequent impact on temporal misperception.

## Data Availability

The datasets generated for this study are available on request to the corresponding author.

## Ethics Statement

This study was carried out in accordance with the recommendations of and approved by the Department of General Psychology, University of Padova (Italy), ethics committee. Written and informed consent was collected from all participants, and the study was conducted in accordance with the Helsinki Declaration (59th WMA General Assembly, Seoul, Republic of Korea, October 2008). Patients with a diagnosis of MCI were recruited from the San Bortolo Hospital, Vicenza, Italy, whereas controls were tested in their own home in the areas of Vicenza and Padova (Italy). Exclusionary criteria for controls included possible dementia or severe cognitive impairment (as defined by MMSE or MoCA scores below the cutoff), whereas treatment with anticholinergic medications, treatment with certain dopaminergic or benzodiazepine medications known to interfere with cognitive functioning, history of neurosurgery or other neurological conditions, significant history or current psychiatric disorders, and any condition (e.g., depression) that would interfere with testing were exclusion criteria for all participants.

## Author Contributions

GM planned and conducted the study. FS and GM did the analyses. FP, MM, and LM helped with neuropsychological evaluation. All authors planned the study and helped in writing the manuscript.

## Conflict of Interest Statement

The authors declare that the research was conducted in the absence of any commercial or financial relationships that could be construed as a potential conflict of interest.

## Footnotes

1 A full description of each neuropsychological tasks can be found in Mioni et al. (2016b) or by consulting the reference for each task.

2 Preliminary analyses were conducted to control for the effect of gender of participants and stimuli. Neither main effect of gender of participants or gender of stimuli nor interactions were found (*p* > 0.05).

3 Inspection of Figure 1B might suggest the presence of an outlier in MCI (shame). Repeated-measures ANOVA was conducted removing that subject to control the results (control = 14, MCI = 11). Analyses of WR showed that no main effects of *Group* (*p* = 0.366, $\eta_{\text{p}}^{2}$ = 0.04; MCI = 0.17, control = 0.15), *Emotion* (*p* = 0.173, $\eta_{\text{p}}^{2}$ = 0.07), or interaction were found (*p* = 0.445, $\eta_{\text{p}}^{2}$ = 0.03).

## Supplementary Material

TABLE S1 Time performance.

TABLE S2 Neuropsychological evaluation.
